# Heterologous expression of heat-resistant obscure (Hero) proteins enhances thermotolerance in plants

**DOI:** 10.1016/j.isci.2025.113249

**Published:** 2025-07-30

**Authors:** Zhe Kong, Yongping Ke, Heng Zhang, Daisuke Miki

**Affiliations:** 1 Shanghai Center for Plant Stress Biology, CAS Center for Excellence in Molecular Plant Sciences, Chinese Academy of Sciences, Shanghai 200032, P.R. China; 2 University of Chinese Academy of Sciences, Beijing 100049, P.R. China; 3 Department of Genetics and Developmental Science, School of Life Sciences and Biotechnology, Shanghai Jiao Tong University, Shanghai 200240, P.R. China; 4 SUAT Institute of Emerging Agricultural Technology, Shenzhen University of Advanced Technology, Shenzhen, Guangdong 518107, P.R. China; 5 Kihara Institute for Biological Research, Yokohama City University, Yokohama, Kanagawa 244-0813, Japan

**Keywords:** Natural sciences, Plant biochemistry, Plant biology, Plant ecology

## Abstract

Improving plant stress tolerance and crop productivity is critical. Membraneless biomolecular condensates formed via liquid-liquid phase separation (LLPS) have been shown to mediate plant responses to various stresses, including heat. While recent advances have elucidated the LLPS role in stress tolerance mechanisms, translating these findings into practical strategies for enhancing plant resilience remains a formidable challenge. In this study, we investigated the effects of two human-derived RNA-binding proteins, Heat-resistant obscure 7 (Hero7) and Hero45, on abiotic stress tolerance in plants. When overexpressed heterologously, these proteins appear to enhance thermotolerance. Under heat stress, Hero7 and Hero45 undergo phase transitions consistent with LLPS, which correlates with the formation of larger processing bodies (PBs) and stress granules (SGs). This may contribute to the protection of mRNAs from heat-induced degradation, potentially improving heat resistance. The present findings demonstrate that LLPS modulation can enhance plant thermotolerance, offering a viable strategy for engineering stress-resistant crops.

## Highlights

• Overexpression of Hero proteins enhances abiotic stress tolerance in plants
• Hero proteins promote LLPS formation under stress conditions
• LLPS may serve as a protective mechanism against mRNA degradation
• LLPS has the potential to enhance the plant’s ability to withstand stress

## Graphical abstract

**Figure:**
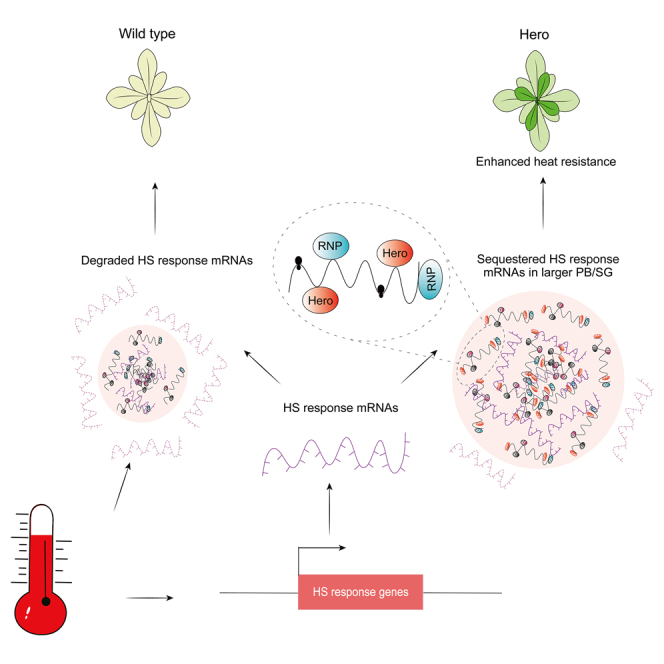

## Introduction

Annually, 30–50% of the world’s principal crops are lost as a consequence of biotic and abiotic stresses.^1^^,^^2^ The mean temperature of the Earth is increasing year by year.^3^ Heat, drought, and salt stresses are among the primary causes of crop losses.^4^ Consequently, maximizing crop yield is vital for ensuring food security. Regulating multiple stress responses and enhancing plant resilience is one of the essential steps to ensure crop productivity.

In nature, plants are frequently exposed to unfavorable abiotic stresses such as drought, heat, cold, and high salinity soils. Both biotic and abiotic stresses induce various changes in plant and cellular processes.^5^^,^^6^ These adaptive changes represent potential targets for crop improvement. However, the regulation of plant stress responses needs to be coordinated with growth-related pathway.^7^ As a result, enhancing crop resilience often leads to unexpected side effects such as growth defects and decreased productivity.^8^ Another challenge in genetic improvement is the complexity of stress response networks. Under natural conditions, plants are often subjected to multiple stresses simultaneously. Therefore, plants genetically modified to enhance specific stress response pathways under laboratory conditions may not survive in the field.^9^ It is therefore necessary to develop a new strategy to enhance comprehensive stress tolerance without compromising plant growth.

Due to the sessile nature of plants, they require highly sensitive and rapid strategies to respond to environmental temperature fluctuations, among which membraneless condensates formed primarily through liquid-liquid phase separation (LLPS) play a crucial role.^10^^,^^11^^,^^12^ Within these condensates, processing bodies (P-bodies: PBs) and stress granules (SGs) are particularly significant.^13^^,^^14^ SGs and PBs function as conserved ribonucleoprotein (RNP) cytoplasmic condensates that regulate mRNA storage, degradation, and translation under various stress conditions, thus maintaining and safeguarding cellular integrity.^15^^,^^16^^,^^17^ Recent evidence increasingly supports the value of LLPS-driven membrane-less condensates in plant heat tolerance. For instance, two functionally redundant RNA-binding proteins RNA-binding glycine-rich D2 (RBGD2) and RBGD4, have been shown to protect heat-responsive transcripts by forming SGs under heat stress through LLPS.^18^ Additionally, another study indicated that Acetylation lowers binding affinity (ALBA4/5/6) proteins can also form P-bodies and SGs via phase separation, protecting heat shock factor (HSF) mRNAs from degradation by the exonuclease 4 (XRN4).^19^^,^^20^ These findings underscore the critical role of P-bodies and SGs in preserving HSF and heat shock protein (HSP) mRNAs during thermal stress.

Heat-resistant obscure (Hero) was initially identified as a novel highly charged and disordered protein in human HEK293T cells. They act as molecular shields, protecting various proteins from denaturation under extreme conditions.^21^ When Hero proteins are mixed with client proteins, Hero can protect the activity of various client proteins under conditions such as heat shock, evaporation, and exposure to organic solvents. This protein-stabilizing capability significantly surpasses that of traditional protein stabilizers, such as arginine and HSPs. Hero proteins function as molecular chaperones.^21^ The primary sequences of HSPs are highly conserved across species, but Hero proteins are specific to the animal kingdom and do not exist in plants. Additionally, chaperone proteins typically act on their target proteins in their inactive state and actively reverse them to their functional state using ATP. However, Hero proteins can influence the functional state of their client proteins and protect them from inactivation and denaturation in an ATP-independent manner.^21^ Six Hero proteins have been identified in animals. Overexpression of Hero proteins in fruit flies has been shown to extend lifespan without detecting any growth defects. Based on these findings, we postulated that heterologous overexpression of Hero proteins may serve to enhance stress tolerance in plants.

To ascertain the veracity of this hypothesis, an investigation was conducted into the biological function of Hero proteins in *Arabidopsis thaliana* (Arabidopsis) and rice. Our findings indicate that Hero7 and Hero45 are capable of forming unusually large PBs and SGs under heat shock conditions. Moreover, we observed that following heat shock, the levels of key mRNAs in the heat response pathway were higher in plants expressing Hero7 and Hero45. We propose that cytoplasmic granules containing Hero proteins can recruit and protect an increased number of mRNAs, thereby enhancing the plant’s heat tolerance.

## Results

### Overexpression of Hero7 and Hero45 increased abiotic stress tolerance without affecting plant growth

Hero proteins, which have been identified as novel, highly charged, and disordered proteins lacking homologous genes in plants, exhibit remarkable heat resistance, high charge, and hydrophilicity.^21^ These properties afford them the ability to safeguard a variety of proteins from denaturation in the face of extreme conditions. Given these characteristics, our objective was to extensively express Hero proteins in plants to gain insight into their role in abiotic stress conditions. Initially, the constitutive strong parsley PcUbq4 promoter with a transcriptional enhancer Arabidopsis AtUbq10 5′ UTR was employed to drive Hero expression in the Col-0 background.^22^^,^^23^^,^^24^^,^^25^ This resulted in six distinct Hero-overexpressing transgenic plants, designated Hero7, 9, 11, 13, 20, and 45-OE, respectively (Figure S1A). Subsequently, the gene expression levels of each Hero gene were evaluated through qPCR (Figure S1B), and the two lines exhibiting the highest expression levels from each Hero gene were selected for further investigation.

**Figure 1:**
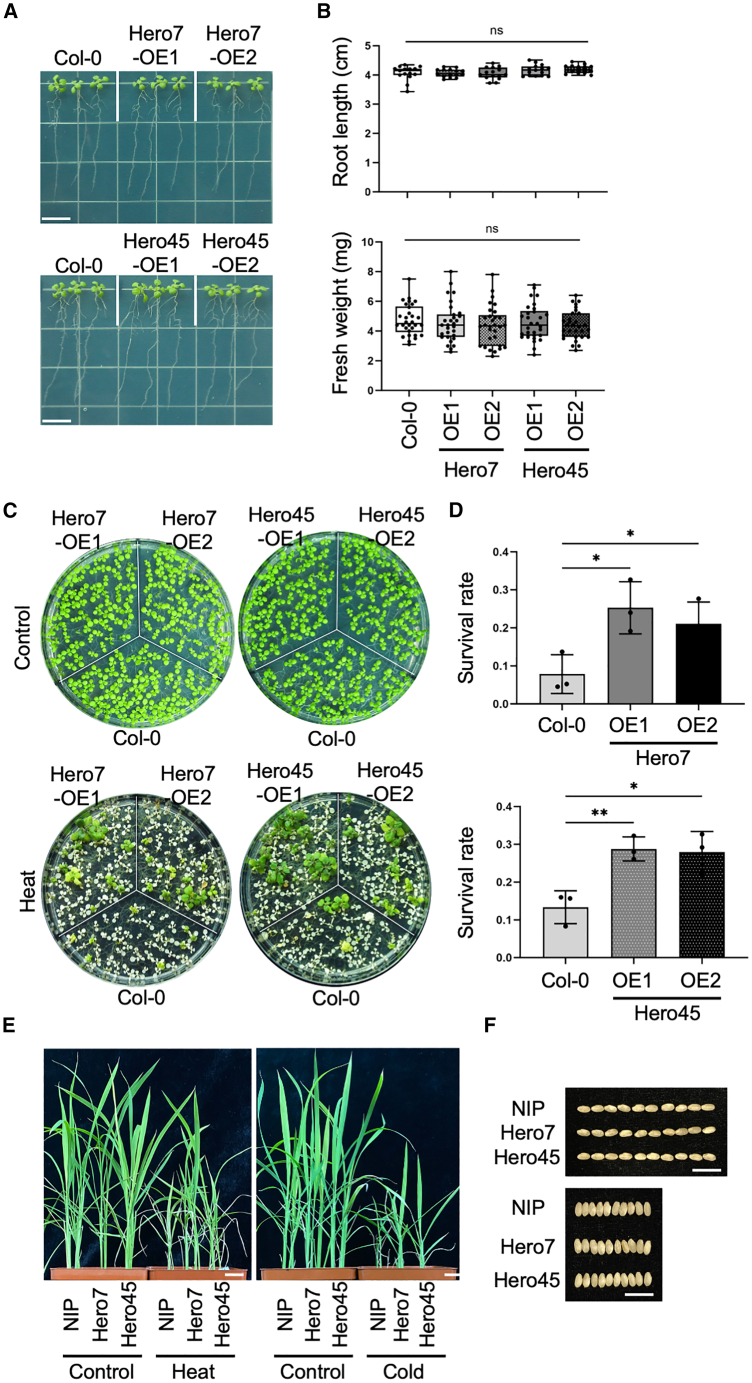
Increased stress tolerance in overexpression of Hero7 and Hero45 (A) Morphological phenotype of Hero7 and Hero 45 Arabidopsis seedling. The plant growth phenotype of 9-day-old Arabidopsis seedlings of the Col-0, Hero7, and Hero45 was analyzed. The plants were grown vertically under normal conditions, and images were taken. Scale bar = 1 cm (B) Quantified analysis of Hero plants. The root length (*n* = 15) and fresh weight (*n* = 30) of 9-day-old Col-0, Hero7, and Hero45 Arabidopsis seedlings were quantified. (C) Hero proteins-mediated heat stress tolerance. The phenotype of 7-day-old Arabidopsis seedlings of the Col-0, Hero7, and Hero45 genotypes under normal conditions (upper panels) and upon exposure to a heat stress treatment (lower panels) is presented. (D) Statistical analysis of the survival ratio. The survival rates of Col-0, Hero7, and Hero45 seedlings following heat treatment were calculated. A Student’s *t* test was employed to determine the standard deviation (*n* = 3). (B and D) Statistical significance with regard to the control sample is indicated: ∗*p* < 0.05, and ∗∗*p* < 0.01. (E) Hero proteins-mediated abiotic stress tolerance in rice. The rice phenotypes of Nipponbare (NIP), Hero7, and Hero45 have been examined under various stress conditions, which have included heat and cold stress treatment. Scale bar = 2 cm. (F) The rice grain phenotype in overexpressing Hero7 and Hero45. The rice grain phenotypes of Nipponbare (NIP), Hero7, and Hero45 were examined under normal growth conditions. Scale bar = 1 cm.

In accordance with the established criteria for normal growth, all plants exhibiting overexpression of the Hero protein demonstrated no observable abnormalities (Figure 1A). The measurements of fresh weight and root length exhibited no significant disparities when compared to the Col-0 control (Figures 1A and 1B). Furthermore, the germination ratio, bolting time, plant height, flower, and silique phenotype of the Hero protein overexpressing plants cultivated under normal growth conditions demonstrate comparable characteristics to those of the Col-0 plants (Figure S2). The results of the experiment demonstrate that the application of Hero did not have a significant impact on the normal growth and development of the plant.

To assess the stress tolerance of the Hero plants, we subjected 7–9-day-old seedlings to a short-term severe heat shock (44°C for 2 h) and allowed them to recover at room temperature (22°C) for 7–10 days, calculating survival rates (Figure 1C). Following the heat treatment, the survival rates of Hero7 and Hero45 seedlings were markedly elevated in comparison to those of Col-0 (Figure 1D), whereas the survival rates of Hero9, 11, 13, and 20 were found to be comparable to those of Col-0 (Figure S3). Additionally, the impact of mannitol stress was also investigated. Although root length remained comparable following treatment with 300 mM mannitol, the chlorophyll content of Hero45-OE seedlings exhibited a marked higher level than that observed in Col-0 seedlings (Figure S4).

Subsequently, the impact of stress-inducible expression of Hero was examined. The RD29A promoter, which is a strong abiotic stress-inducible gene, was used in conjunction with the AtUbq10 5′ UTR to drive Hero7 (Figure S5A). The stress-inducible expression of Hero7 resulted in comparable survival rates under heat stress conditions to those observed in Col-0, yet lower than those observed in Hero7-OE (Figure S5B). On the other hand, the chlorophyll content of stress-inducible Hero7 and Hero7-OE plants was higher than that observed in Col-0 seedlings under mannitol treatment conditions (Figure S5C).

Additionally, an investigation was conducted to ascertain whether the localization of Hero45 within the chloroplast membrane could enhance the protection of chloroplasts by fusing a signal peptide derived from sHSP21 with Hero45. However, the chloroplast membrane-localized overexpression of Hero45 did not result in an improvement in plant heat tolerance (Figure S6). These findings suggest that the constitutive expression of Hero proteins, and not their localization at the chloroplast membrane, should be employed to enhance abiotic stress tolerance in plants.

In order to gain insight into the broader implications, the impact of heterologous overexpressing the Hero proteins in rice on abiotic stress tolerance was investigated. Transgenic rice (*Oryza sativa*) plants were developed through constitutive overexpression of the Hero proteins, driven by the maize (*Zea mays*) ZmUbq1 promoter.^26^ As observed in Arabidopsis, heterologous overexpression of Hero7 and Hero45 in rice plants resulted in robust tolerance to heat and cold stress (Figure 1E). Moreover, the growth characteristics, including productivity and grain size, of these Hero transgenic rice plants were found to be comparable to those of the Nipponbare control under normal growth conditions (Figures 1E and 1F). Consequently, it can be concluded that the heterologous overexpression of Hero7 and Hero45 enhances stress tolerances, such as heat, without affecting normal growth in plants.

### Reversible localization of Hero proteins to cytoplasmic granules under heat stress

**Figure 2:**
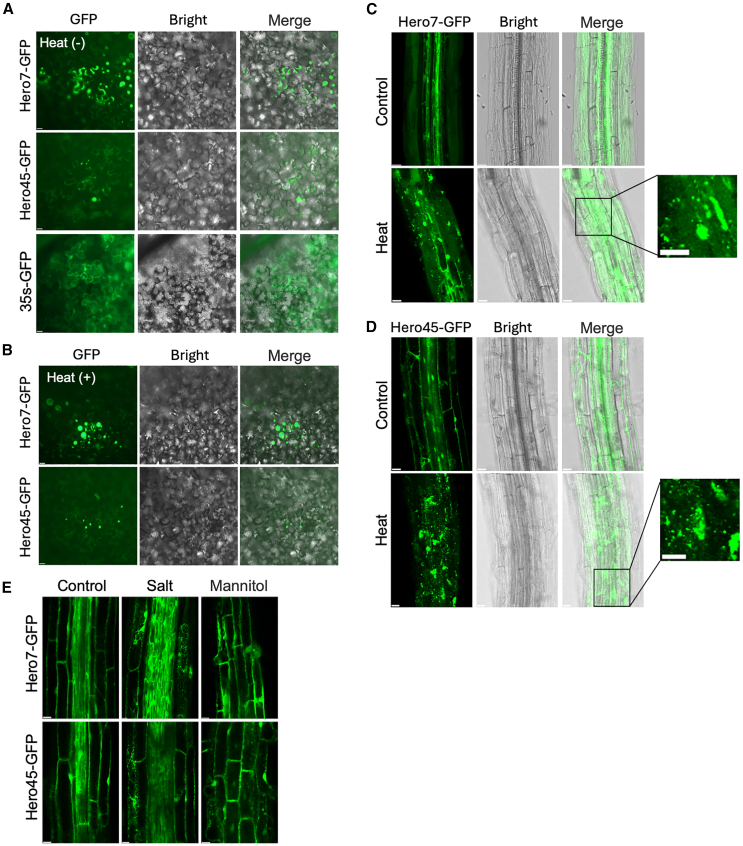
Localization of Hero7 and 45 to cytoplasmic granules under stress conditions (A and B) Subcellular localization of the Hero proteins in tobacco leaves. Fluorescence images demonstrate the localization of Hero7-GFP, Hero45-GFP, and 35s-GFP in tobacco under normal (A) and heat stress (42°C for 50 min) (B) conditions. Scale bar = 10 μm. (C and D) Subcellular localization of the Hero proteins in Arabidopsis. Fluorescence images demonstrate the localization of Hero7-GFP (C) and Hero45-GFP (D) in Arabidopsis root tissue under control conditions and heat stress (42°C for 50 min). Scale bar = 5 μm. (E) Subcellular localization of the Hero proteins in response to salt and osmotic stress. Fluorescence images demonstrate the localization of Hero7-GFP and Hero45-GFP in Arabidopsis under conditions of salt stress (100 mM NaCl) and osmotic stress (300 mM mannitol) for a period of 2 h. Scale bar = 5 μm.

The molecular mechanisms by which Hero proteins contribute to thermostability in plants were investigated. The Hero7 and Hero45 proteins were fused with Green Fluorescent Protein (GFP) and transiently expressed in tobacco leaves of the *Nicotiana benthamiana* plant by Agrobacterium infiltration. The 35S-GFP was employed as a control for subcellular localization studies. At a normal growth temperature of 22°C, both the Hero7-GFP and Hero45-GFP exhibited a diffused distribution within the cytoplasm, with a minor presence in the nucleus (Figure 2A). In addition, both proteins formed spherical granules localized in the cytoplasm. Following heat treatment at 42°C for 50 min, the signals for Hero7-GFP and Hero45-GFP aggregated in the cytoplasm, and the spherical cytoplasmic granules increased in size and number (Figure 2B).

In order to ascertain whether this aggregation in tobacco leaves is associated with thermo-stress tolerance, transgenic Arabidopsis lines expressing Hero7-and Hero45-GFP in a wild-type Col-0 background were obtained. The subcellular localization of Hero7-GFP and Hero45-GFP in the root tips of 7-day-old seedlings was examined. As observed in tobacco, under normal growth conditions, the signals of Hero7-GFP and Hero45-GFP were distributed throughout the cytoplasm and the nucleus, though they rarely formed spherical granules. Following a 50-min heat treatment at 42°C, numerous granular structures were observed in the Hero7 and Hero45 plants (Figures 2C and 2D).

Further investigations were conducted on the formation of spherical granules by Hero7 and Hero45 under various types of abiotic stress. After treating with salt stress (100 mM NaCl) and osmotic stress (300 mM mannitol) for 2 h, Arabidopsis root cells similarly formed spherical granules (Figure 2E). This suggests that Hero7 and Hero45 proteins can serve as a general component of spherical granules, participating in their formation under various abiotic stress conditions. These results are consistent with our observed phenotypes, leading us to speculate that Hero proteins may improve plant stress tolerance by influencing phase separation.

### Hero proteins-mediated liquid-liquid phase separation in plants

**Figure 3:**
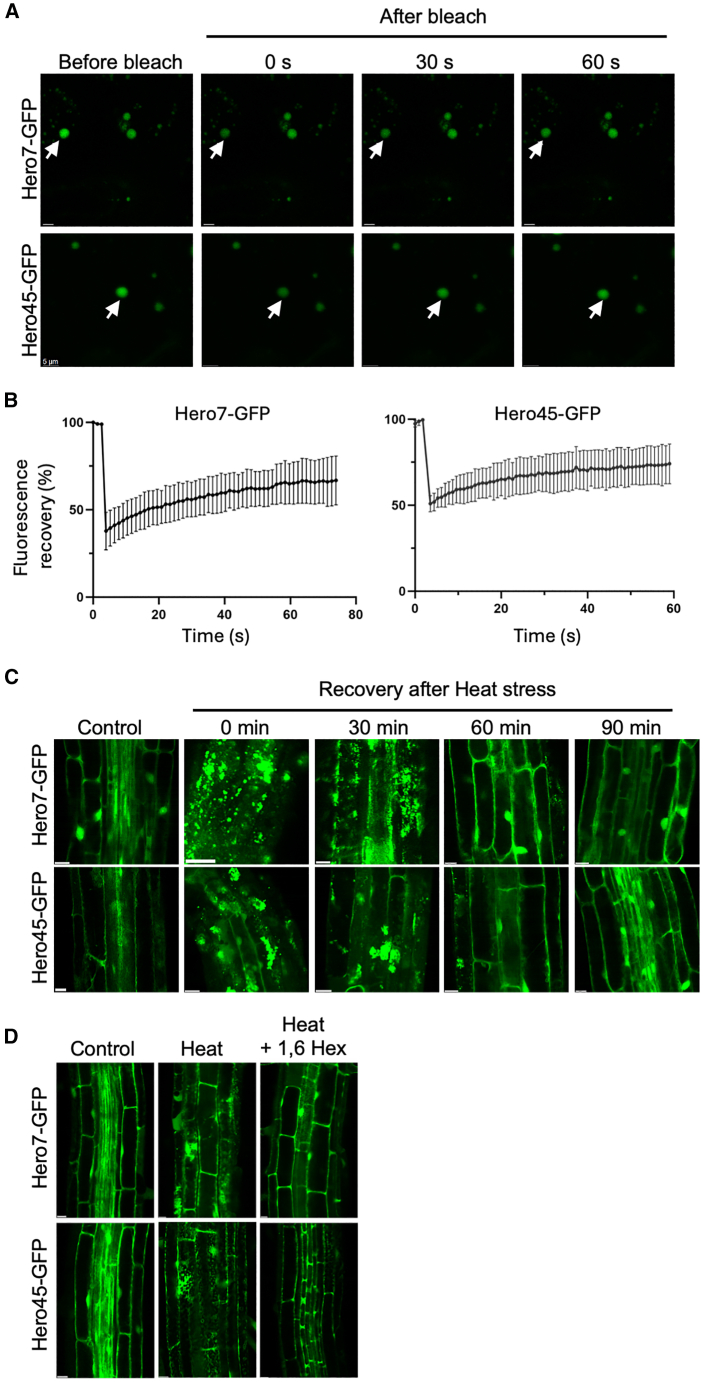
Liquid-liquid phase separation via Hero7 and Hero45 in plants (A) FRAP analysis of Hero granules. FRAP of Hero7-GFP and Hero45-GFP granules in the cytoplasm of tobacco leaf cells. The white arrows indicate regions where fluorescence has been reduced. Scale bar = 5 μm. (B) Statistical analysis of FRAP. The fluorescence recovery curve of Hero7-GFP and Hero45-GFP cytoplasmic granules after photobleaching was quantified. Data are presented as mean ± SD (*n* = 4). (C) Subcellular localization of Hero proteins during the recovery phase. The fluorescence images demonstrate the localization of Hero7-GFP and Hero45-GFP during the recovery phase from heat stress. Scale bar = 10 μm. (D) Subcellular localization of Hero proteins with LLPS inhibitor. Fluorescence images demonstrate the localization of Hero7-GFP and Hero45-GFP under heat stress conditions with and without the treatment of LLPS inhibitor 1,6-hexanediol. Scale bar = 10 μm.

A key question that warrants investigation concerns the nature of granular structures generated under stress conditions by both the Hero7 and Hero45 proteins, as well as the potential mechanisms involved. It has been documented that the formation of membrane-free biomolecular condensates by LLPS under stress conditions in plants has been observed.^18^^,^^19^^,^^27^^,^^28^^,^^29^ It is therefore postulated that the formation of spherical cytoplasmic granules in both tobacco and Arabidopsis indicates the occurrence of membraneless biomolecular condensate formation driven by LLPS. The objective was to ascertain if Hero7 and Hero45 is capable of undergoing LLPS within plant cells. Initially, fluorescence recovery after photo bleaching (FRAP) was employed to investigate the dynamics of granules containing Hero proteins in tobacco leaves transiently expressing Hero7-GFP and Hero45-GFP. The granules with a radius of approximately 2 μm, which were induced by heat stress, were selected for photobleaching, and the rapid fluorescence recovery was observed within 60–90 s post-bleaching (Figures 3A and 3B). This finding indicates that the granules formed by Hero7 and Hero45 exhibit liquid-like properties. Therefore, it is reasonable to hypothesize that the granular structures produced under stress by Hero7 and Hero45 may be regarded as LLPS.

Subsequently, the reversibility of the granule structures formed by the Hero proteins was investigated. The Arabidopsis seedlings that had been subjected to heat treatment were returned to temperatures conducive to normal growth in order to facilitate recovery. In the initial 30 min following heat treatment, the number of granules in the cells remained high; however, a notable decline was observed after 60 min, with granules largely disappearing by approximately 90 min (Figure 3C). This indicates that heat-induced granules possess the capacity for plasticity *in vivo*.

To further assess the potential involvement of LLPS in the formation of these granular structures, root tip cells of heat-stressed seedlings were treated with 2% 1,6-hexanediol for a period of 5 min. The chemical 1,6-hexanediol, which has been shown to disrupt LLPS structures through moderate hydrophobic interaction interference without damaging biomembrane structures,^30^ was observed to effectively abolish the formation of Hero granules (Figure 3D). This observation lends further credence to the hypothesis that LLPS contributes to Hero granule formation.

Furthermore, the potential of the Hero7 and Hero45 to function as a scaffold for facilitating LLPS was investigated. Scaffold proteins that drive LLPS are known to contain long intrinsically disordered regions (IDRs).^31^ The DisoPred algorithm was employed to analyze the sequence of Hero7 and Hero45, with the GFP sequence serving as a control due to the observed formation of spherical granules when fused to GFP. The results obtained from this analysis were consistent with the original research on Hero proteins,^21^ indicating that the full sequence of Hero7 and Hero45 is predicted to be intrinsically disordered, in contrast to the sequence of GFP (Figure S7). The probability of forming phase separation is directly proportional to the length of the disordered sequence. Consequently, these observations are consistent with the possibility that Hero7 and Hero45 may undergo LLPS *in vivo*, potentially contributing to the formation of granules with liquid-like properties and reversibility.

### Localization of Hero7 and Hero45 to PBs and SGs under heat stress in plants

**Figure 4:**
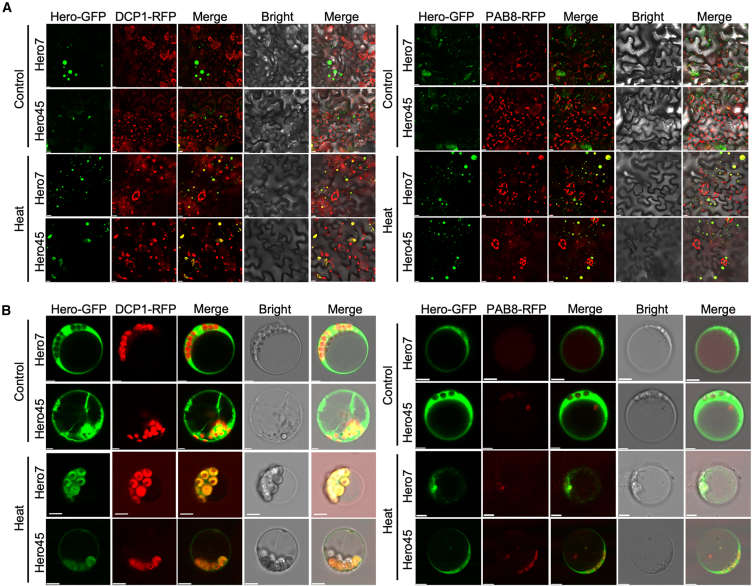
Localization of Hero7 and Hero45 proteins to SG and PB under heat stress in tobacco and Arabidopsis (A and B) Subcellular localization of Hero proteins. Fluorescence images demonstrating the colocalization of Hero7-GFP and Hero45-GFP with PB marker proteins DCP1-RFP and SG marker proteins PAB8-RFP under normal and heat stress conditions in tobacco leaves Scale bar = 10 μm (A) and in Arabidopsis protoplasts Scale bar = 5 μm (B).

In order to determine whether the observed reversible cytoplasmic granules were P-bodies (PB) or stress granules (SG), the co-localization of Hero7 and Hero45 with marker proteins was examined. The poly(A) binding protein 8 (PAB8) and the Decapping 1 (DCP1) were utilized as SG and PB marker proteins, respectively.^32^^,^^33^ The transient expression of Hero7-and Hero45-GFP in conjunction with DCP1- and PAB8-RFP in tobacco resulted in the observation that, under normal growth conditions, although Hero7 and Hero45 were capable of forming droplet-like granules, they did not coexist with PB and SG marker proteins. However, upon exposure to heat stress, colocalization of Hero7 and Hero45 with both PB and SG markers was observed (Figure 4A).

A comparable transient expression assay was performed in Arabidopsis protoplasts. The results were analogous to those observed in tobacco leaves, wherein Hero7 and Hero45 colocalized with both PAB8 and DCP1 following heat stress (Figure 4B). In conclusion, the results of the present study indicate that the Hero7 and Hero45 proteins are capable of forming PBs and SGs in plants subjected to heat stress conditions.

### Elevation of heat stress response-related mRNAs in the Hero plants

**Figure 5:**
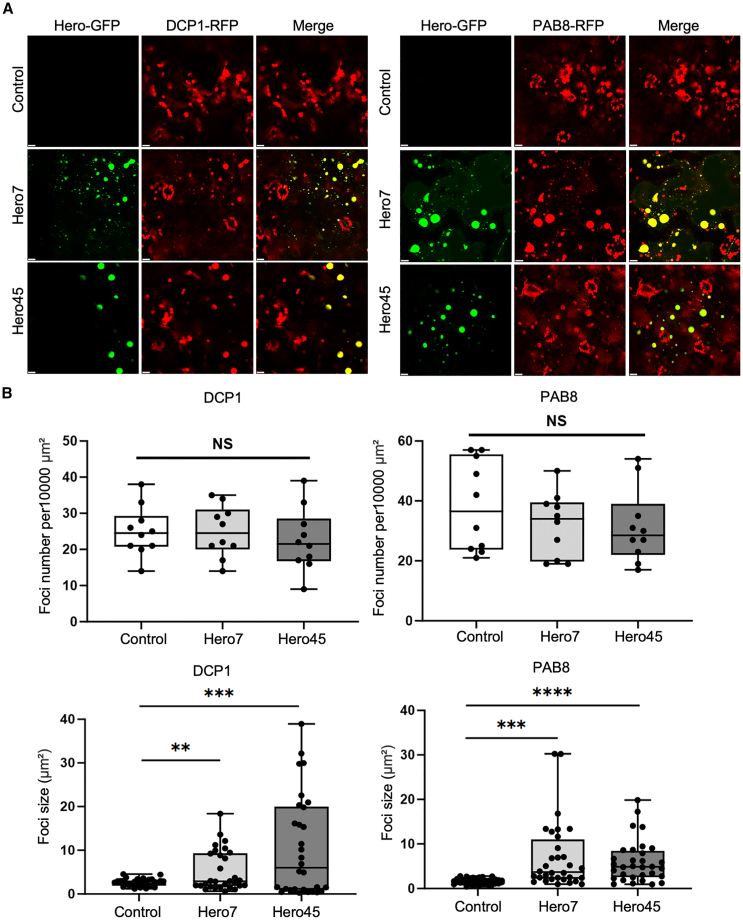
Increased size of SGs and PBs in Hero7 and Hero45 plants (A) Subcellular localization of Hero proteins under heat stress. Fluorescence images demonstrate the colocalization of Hero7-GFP and Hero45-GFP with PB marker proteins DCP1-RFP and SG marker proteins PAB8-RFP under both normal and heat stress conditions in tobacco. Scale bar = 10 μm. (B) Quantitative analysis of granule number and size. A quantitative analysis of the number (*n* = 10) and size (*n* = 30) of granules in control tobacco leaves, as well as in leaves expressing Hero7-GFP and Hero45-GFP, was conducted. Statistical significance with regard to the control sample is indicated: ∗∗*p* < 0.01, ∗∗∗*p* < 0.005, and ∗∗∗∗*p* < 0.001.

In light of our findings that Hero7 and Hero45 undergo LLPS, and localizes to SGs and PBs in response to heat stress, we sought to investigate the role of Hero proteins in phase separation and their potential to enhance plant thermotolerance. The fusion proteins Hero7-and Hero45-GFP with DCP1- or PAB8-RFP were transiently expressed in tobacco leaves. Following the application of the heat stress treatment, the number of SGs and PBs formed in the tobacco leaf expressing the Hero proteins was found to be comparable to that observed in the control plants (Figure 5). In the control groups, the sizes of the SGs and PBs were observed to be generally consistent with one another. In contrast, while some SGs and PBs containing Hero proteins were similar in size to the control, others were markedly larger than those observed in the control group. Moreover, the dimensions of SGs lacking Hero protein localization in tobacco leaves were analogous to those of SGs in the control group, and PBs exhibited a comparable size pattern to SGs (Figure 5). These findings suggest that Hero proteins have a distinctive function in the formation of SGs and PBs in plants.

**Figure 6:**
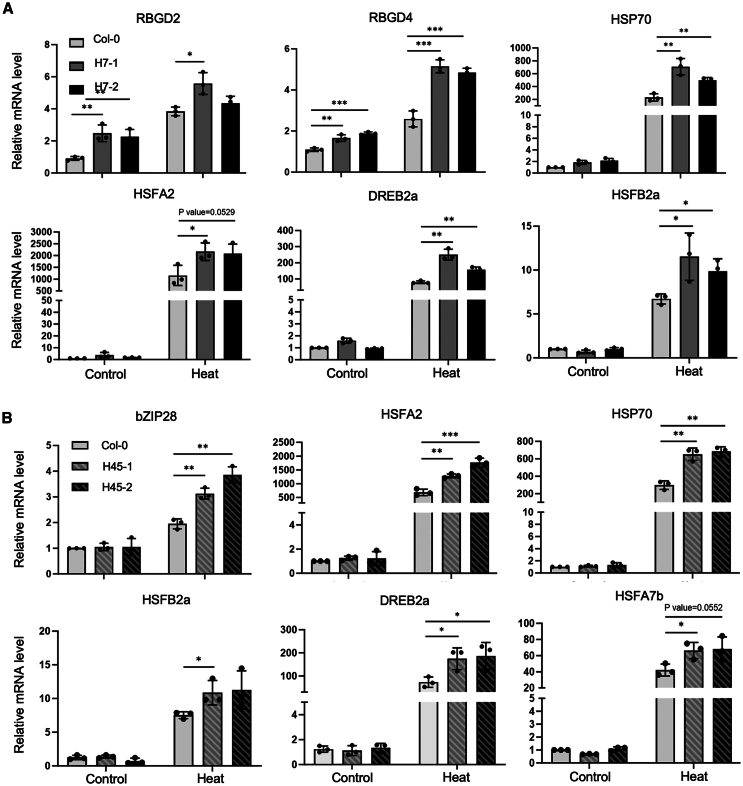
Elevated heat stress response-related mRNAs by LLPS in Hero7 and Hero45 plants (A and B) Quantitative mRNA expression analysis. The relative mRNA levels of the indicated genes in Col-0, Hero7-OE (A), and Hero45-OE (B) plants were investigated under both normal and heat stress (44°C for 2 h) conditions using qRT-PCR. The *AtAct7* were utilized as an internal control for qRT-PCR. The data presented here represent the mean ± standard deviation of three biological replicates. (∗*p* < 0.05, ∗∗*p* < 0.01).

Given the crucial role of SGs and PBs in mRNA storage,^19^^,^^34^ we postulated that the increased size allows SGs and PBs to recruit a large number of mRNA molecules, thereby protecting them from degradation under heat stress. Subsequently, samples were collected from 7-day-old Hero7 and Hero45 plants and corresponding age Col-0 seedlings after 100 min of heat treatment at 44°C. The mRNA content of these samples was then analyzed by qRT-PCR. The mRNA levels of key heat-responsive genes, including *HSP70*, *HSFA2*, *DREB2a*, and *HSFB2a*, were found to be significantly elevated in the Hero7 and Hero45 plants relative to Col-0 following heat treatment (Figure 6). Moreover, mRNA levels of *HSFA7B* and the endoplasmic reticulum stress pathway gene *BZIP28* were also elevated post-heat stress in Hero45 compared to Col-0. It is noteworthy that the mRNA levels of *RBGD2* and *RBGD4* were found to be elevated in Hero7 relative to Col-0, under both normal conditions and following heat stress (Figure 6A). These findings are consistent with the augmented thermotolerance observed in Hero7 and Hero45. In summary, the present findings suggest that Hero proteins contribute to the formation of PBs and SGs, promoting the recruitment and stabilization of multiple mRNA copies, which may support improved thermotolerance in plants.

## Discussion

In order to enhance plant stress tolerance, a number of genetic modifications have been attempted, including mutagenesis and the overexpression of endogenous or exogenous genes.^5^^,^^6^^,^^35^^,^^36^ However, these genetic modifications frequently impact specific pathways and often result in unanticipated adverse side effects, such as growth defects and diminished productivity.^7^^,^^37^ The use of animal genes that lack homologous genes in plants as exogenous genes to enhance plant stress tolerance is a relatively common practice. There is a paucity of literature reporting the application of human genes in plants. A previous study indicated that the Fat mass and obesity-associated (FTO) protein, which mediates the demethylation of human RNA (m6A), led to increased yield and biomass in rice and potato.^38^ This result suggests that, as a heterologous protein, FTO may not be recognized or regulated by plant components in the conventional manner, potentially influencing multiple targets and resulting in unexpected effects.

It is noteworthy that, despite the absence of homologous genes in plants, the functions of Hero proteins do not deviate from traditional mechanisms. Indeed, mouse-derived Hero7 and Hero45 have been demonstrated to participate in stress granule (SG) formation in mouse cells.^39^^,^^40^ For example, Hero45 has been shown to interact with the core scaffold protein G3BP1 in stress granules (SGs), which is highly conserved in both animals and plants. It is therefore supposed that Hero7 and Hero45 may function by interacting with conserved scaffold proteins in plant SGs and PBs. Given that the remaining four Hero proteins have not been previously implicated in phase separation, it is hypothesized that these proteins may lack the capacity to engage in this process in plants. Consequently, they may be unable to enhance heat resistance in plants under the experimental conditions employed.

Upon initial discovery, it was considered that Hero proteins represented a novel molecular shield, capable of protecting client proteins from aggregation.^21^ Our initial hypothesis was to express as many Hero proteins as possible in plants to fully utilize their chaperone functions, thereby preventing intracellular protein aggregation under extreme conditions. However, when comparing the total number of denatured proteins in Hero7 and Hero45 plants, we found that the results did not correlate with the observed phenotypes. This led us to hypothesize that the Hero7 and Hero45 proteins may also enhance plant stress resistance through alternative mechanisms from the original discovery.

**Figure 7:**
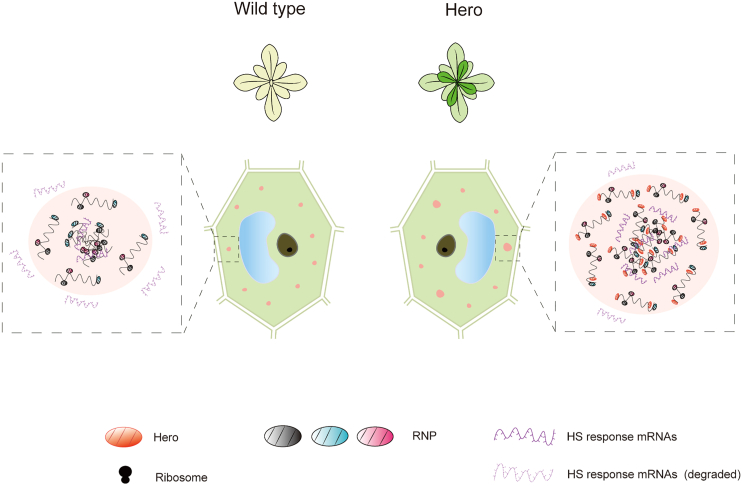
Hypothetical working model of Hero proteins under abiotic stress conditions The diagram illustrates a hypothetical functional model for the role of the Hero protein in the heat stress response in plants. In the presence of heat stress, the heterologous Hero protein expression plants manifest an increase in the size of the droplets (depicted in pink) within their cells. This mechanism facilitates the recruitment of additional mRNA (dark purple) into processing bodies (PBs) and stress granules (SGs) through phase separation, thereby safeguarding the mRNA from degradation (light purple).

Upon analysis of the amino acid sequences of these two proteins, it was determined that Hero7 and Hero45 are composed entirely of intrinsically disordered proteins (IDPs). It is postulated that these regions facilitate homotypic molecular interactions, which serve as the thermodynamic driving force for phase separation. This is the inaugural report to indicate that Hero7 and Hero45 have the potential to undergo LLPS in plants and colocalize with the PBs and SGs under heat stress conditions. Notably, the sizes of SGs and PBs containing Hero proteins were found to be significantly larger than those lacking Hero proteins. Given that the dimensions of SGs and PBs lacking Hero proteins were nearly indistinguishable, it is plausible that the incorporation of Hero proteins could facilitate the assembly of PBs and SGs in plants (Figure 7). In response to heat stress, the initial formation of PBs and SGs is dependent on the recruitment of proteins and mRNAs by endogenous, IDR-rich scaffold protein.^19^^,^^41^^,^^42^ It is hypothesized that, over time, the high-concentration core regions composed of proteins and mRNAs may stabilize, while the surrounding, more loosely structured “shell” continues to exchange components with the external environment. This exchange may potentially limit further growth of PBs and SGs. The incorporation of Hero proteins has been posited as a potential solution to this challenge. It is hypothesized that, given their structural features, including IDRs and RNA-binding domains, Hero proteins may interact with other endogenous IDR-rich scaffold proteins and mRNAs within PBs and SGs (Figure 7). This interaction could, over time, contribute to the formation of larger PBs and SGs, possibly enhancing mRNA recruitment and protection from degradation.

Hero7 and Hero45 are newly identified IDPs in human cells,^21^ and despite lacking common or conserved domains, they exhibit similar functions in plants according to present research. We observed several molecular characteristics shared by Hero7 and Hero45, such as unusual heat resistance, flexible structures, high charge, and a composition rich in hydrophilic amino acids. These characteristics bear resemblance to those observed in the Late Embryogenesis Abundant (LEA) protein family, which is widely present within the plant kingdom.^43^^,^^44^ Most LEA proteins have molecular weights ranging from 10 to 30 kDa, similar to Hero proteins, and they exhibit high heat resistance, hydrophilicity, and long internal disordered regions.^45^ Multiple studies have shown that overexpression of LEA proteins, which primarily localize in the cytoplasm and nucleus, enhances plant resistance to drought, high salinity, and temperature stresses without significantly affecting normal growth.^45^^,^^46^^,^^47^^,^^48^ Given the numerous similarities between these proteins, we hypothesize that Hero proteins likely function similarly to LEA proteins under normal growth conditions. Due to their high hydrophilicity and extensive disordered regions, Hero proteins exist in an unfolded state in aqueous solutions under normal conditions, making it challenging for them to interact with other proteins. However, under specific induction conditions such as extreme temperatures or dehydration, Hero proteins may form a high proportion of α-helices, thereby gaining functional capabilities.^45^^,^^49^ This could explain why overexpression of Hero proteins does not affect plant growth under normal conditions.

Interestingly, LEA proteins have long been regarded as intracellular molecular chaperones that form complexes with client proteins to prevent their misfolding.^50^^,^^51^ However, recent studies have revealed that LEA proteins also participate in phase separation. For instance, LEA7 and LEA29 can form droplet-like cellular granules in tobacco, and heterologous expression of AfrLEA6 (AWM11684) in insect cells enhances desiccation tolerance through the formation of cytoplasmic MLOs (membraneless organelles).^30^^,^^52^ In light of the aforementioned reports and the findings of the present study, it can be posited that Hero and LEA proteins exhibit a considerable degree of similarity with regard to their molecular characteristics and biological functions.

This study demonstrates that the heterologous overexpression of two human proteins, Hero7 and Hero45, facilitated the improvement of a wide range of abiotic stress tolerances in Arabidopsis and rice plants without trade-offs. The molecular analysis conducted in this study demonstrated that the heterologous expression of Hero proteins promotes the development of larger PBs and SGs under conditions of heat stress in plants. It has been posited that this process may result in the preservation of a greater number of stress response-related mRNAs from degradation (Figure 7). Despite the absence of empirical evidence from the present study on the mechanism in rice, it is hypothesized that the same mechanism would likely be applicable to the tolerance of abiotic stress in a wide range of plants. Further elucidation is required in regard to the molecular mechanisms involved, including whether the heat tolerant phenotype is conferred by their LLPS nature, how Hero7 and Hero45 expression results in elevated heat stress response-related mRNAs in SGs and PBs, and whether Hero proteins directly or indirectly recruit mRNAs into PBs and SGs. In summary, the results of this study suggest that the heterologous expression of Hero proteins has the potential to significantly enhance stress tolerances in plants without any adverse effects. This suggests the emergence of a wide range of potential applications in the future, including the improvement of stress tolerance in economically significant crops.

### Limitations of the study

While this study provides valuable insights into the heterologous overexpression of human Hero proteins and their ability to enhance abiotic stress tolerance in plants without trade-offs, several limitations should be acknowledged. First, the molecular mechanisms underlying the observed increase in SG and PB size remain unclear. A more thorough investigation is necessary to elucidate the mechanisms by which these structures recruit and protect a significant number of mRNA molecules under heat stress. Furthermore, the potential synergistic effects of combining different Hero proteins to enhance stress tolerance have yet to be explored and warrant further investigation. While the findings demonstrate stress tolerance benefits under laboratory conditions, it is imperative to validate these results in outdoor field environments to assess their real-world applicability.

## Resource availability

### Lead contact

Further information and requests for resources and reagents should be directed to and will be fulfilled by the Lead Contact, Daisuke Miki (daisuke.miki@suat-sz.edu.cn).

### Materials availability

• This study did not generate new unique reagents.
• All plasmid and transgenic plant lines generated in this study are available from the lead contact with a completed Materials Transfer Agreement.

### Data and code availability

• All data will be shared by the lead contact upon request after publication.
• No original code was generated in this study.
• Any additional information required to reanalyze the data reported in this work paper is available from the lead contact upon request.

## Acknowledgments

This work was supported by the Shanghai Science and Technology Innovation Plan (20ZR1467000 and 23WZ2500800), the Foreign Expert Project (G202201355L), the Chinese Academy of Sciences, and Shenzhen University of Advanced Technology to D.M. We would like to express our gratitude to Professor Yukihide Tomari at the Institute for Quantitative Biosciences, The University of Tokyo, for kindly sharing their Hero proteins with us. We would like to thank all laboratory members of the Epigenetics and Genome Engineering Group and the Shanghai Center for Plant Stress Biology, CAS Center for Excellence in Molecular Plant Sciences, Chinese Academy of Sciences, and Shenzhen University of Advanced Technology for their assistance.

## Author contributions

Z.K. and D.M. designed the research; D.M. supervised the project; Z.K. performed the experiments with assistance from Y.P.K. and H.Z.; Z.K. and D.M. wrote the paper. All authors contributed to the article and approved the submitted version.

## Declaration of interests

The authors declare no competing interests.

## STAR★Methods

### Key resources table

**Table:** 

REAGENT or RESOURCE	SOURCE	IDENTIFIER
**Chemicals, peptides, and recombinant proteins**		

Cetyltrimethyl ammonium bromide	Sigma	Cat# 57-09-0
Murashige & Skoog (MS) medium	PhytoTech Labs	M519
Sodium chloride	Sigma	S9888
Mannitol	Sigma	MX0214
Hygromycin	Sigma	10843555001
TRIzol	Sigma	T9424

**Critical commercial assays**		

DNA labeling kit	Takara	Cat# 6045
RNeasy Plant mini kit	Qiagen	Cat# 74904
RNase-free Dnase	Qiagen	Cat# 79254
cDNA Synthesis SuperMix kit	TransGen Biotech	Cat# AH301-02
ChamQ SYBR qPCR Master Mix	Vazyme	Cat# Q311-02

**Experimental models: Organisms/strains**		

Arabidopsis Hero overexpression transgenic lines	This paper	N/A
Rice Hero overexpression transgenic lines	This paper	N/A

**Oligonucleotides**		

See Table S1 for all oligonucleotides used in this study		

**Recombinant DNA**		

pCambia1300	Addgene	Cat# 5930
pPcUbq4-Hero	This paper	N/A
pZmUbq1-Hero	This paper	N/A
p35S-Hero7-GFP	This paper	N/A
p35S-Hero45-GFP	This paper	N/A
p35S-DCP1-RFP	This paper	N/A
p35S-PAB8-RFP	This paper	N/A

**Software**		

GraphPad Prism	GraphPad Software	v8, RRID: SCR_002798
ImageJ v 1.53a	NIH	https://imagej.nih.gov/
Leica Application Suite X	Leica Microsystems	RRID: SCR_013673

### Experimental model and subject details

#### Arabidopsis

*Arabidopsis thaliana* (Col-0) for Hero transgenic lines generation was germinated on 1/2 Murashige & Skoog (MS) solid medium and cultured in incubator for two weeks, then transplanted seedlings into soil in the greenhouse. The growth condition is light/16 h and dark/8 h at 22°C.

#### Tobacco

*Nicotiana benthamiana* for Agrobacterium transient assay was germinated on 1/2 Murashige & Skoog (MS) solid medium and cultured in incubator for one-two weeks, then transplanted seedlings into soil in the greenhouse. The growth condition is light/16 h and dark/8 h at 22°C.

#### Rice

The Japonica rice *Oryza sativa* cultivar Nipponbare was used for all experiments. All plants were grown at 28 °C in soil with a 12 h light/12 h dark photoperiod in a greenhouse.

### Method details

#### Gene accession numbers

*DCP1*, At1g08370; *PAB8*, At1g49760; *RBGD2*, At2g33410; *RBGD4*, At4g14300; *HSP70*, At4g16660; *HSFA2*, At2g26150; *DREB2a*, At5g05410; *HSFB2a*, At5g62020; *bZIP28*, At3g10800; *HSFA7b*, At3g63350.

#### Plant materials and growth condition

*Arabidopsis thaliana* (Col-0) for Hero transgenic lines generation was germinated on 1/2 Murashige & Skoog (MS) solid medium and cultured in incubator for two weeks, then transplanted seedlings into soil in the greenhouse. The growth condition is light/16 h and dark/8 h at 22°C.

#### Stress tolerance assay

The heat stress tolerance assays were conducted using 7-day-old seedlings. After vernalization, the seedlings were grown at 22°C for 7 days, then exposed to a growth chamber set at 44°C for 120 minutes, followed by a recovery period at 22°C for 10–14 days. The survival rate was calculated based on the activity of the apical meristem. For the salt stress experiment, seeds were directly placed on 1/2 MS medium containing 100 mM NaCl. In the osmotic stress experiment, seedlings were grown at 22°C for 1–2 days, after which seedlings with similar root lengths were transferred to vertical plates containing 300 mM mannitol. The percentages of seedlings in different phenotypic classes were determined based on the results from three biological replicates. In both the heat and salt stress experiments, each biological replicate consisted of at least 50 seedlings per genotype.

#### Plasmid construction

The constitutive strong parsley PcUbq4 promoter and a transcriptional enhancer Arabidopsis AtUbq10 5′ UTR were employed to drive Hero expression in the Arabidopsis Col-0 background.^22^^,^^23^^,^^24^^,^^25^ Similarly, the constitutive strong maize ZmUbq1 promoter and a transcriptional enhancer Arabidopsis AtUbq10 5′ UTR were employed to drive Hero expression in the rice Nipponbare cultivar background.^26^ The overexpression and GFP fusion constructs were generated in the pCambia1300 background in all cases.

#### Transformation of Arabidopsis plant

The constructed vector was transferred into *Agrobacterium tumefaciens* GV3101 for subsequent transformation experiments in Arabidopsis. All primers used in this study are listed in Table S1.

Arabidopsis was transformed via the Floral Dip method using *Agrobacterium tumefaciens* carrying destination vectors. Transformed T1 generation seeds were screened using the Basta method, in which seeds were spread on soil and 0.02% Glufosinate Ammonium solution (SANGON) was applied three to four times. For hygromycin-based screening, T1 seeds were grown on a 1/2 MS plate containing 50 mg/L hygromycin. Surviving seedlings were transferred to the soil for further cultivation and genotyping.

#### Genomic DNA extraction

The total genomic DNA was extracted from leaf tissue by the cethyltrimethyl ammonium bromide (CTAB) method for individual plant analysis. Leaf tissues were ground into fine powder in liquid nitrogen using ShakeMaster AUTO (Bio Medical Science Inc., Tokyo, Japan). Extracted DNA was used for the following DNA analyses.

#### RNA analysis

Arabidopsis samples were collected from 7-day-old Hero7 and Hero45 plants and control Col-0 seedlings after 100 minutes of heat treatment at 44°C. Seedling tissues were ground into fine powder in liquid nitrogen using ShakeMaster AUTO (Bio Medical Science Inc., Tokyo, Japan). Total RNAs were extracted using the TRIzol (Sigma) method, and RNase-free DNase (Qiagen) was used to remove contaminating DNA before quantitative real-time PCR (qRT-PCR). For qRT-PCR, total RNAs were reverse transcribed with the TransScript One-Step gDNA Removal and cDNA Synthesis SuperMix kit (Transgen) according to the instructions. All qRT-PCR assays were performed following the protocol of the ChamQ SYBR qPCR Master Mix (Vazyme) in a CFX96 Real-time system (BIO-RAD) according to the manufacturer’s instructions. The *AtAct7* were used as internal controls for Arabidopsis in the qRT-PCR. Sequence of primers for qPCR are provided in the Table S1.

#### FRAP assay

In tobacco leaves, FRAP analysis was performed using the FRAP module of a Leica TCS SP8 confocal microscope to acquire fluorescence data. The tobacco was first heat-treated at 42°C for 1 hour to induce particle formation. Hero-GFP signals were excited at 488 nm and detected in the range of 500–540 nm. A moderate-sized particle was chosen and signal stability was assessed by performing 3–5 iterations to ensure signal consistency. Subsequently, particles were bleached five times with a 405 nm laser at 100% intensity. After fluorescence bleaching, images were continuously iterated at 1.29-second intervals until fluorescence recovery was no longer significant. The FRAP module of the Leica Application Suite X (LAS X) was utilized to acquire FRAP data. At each designated time point, the fluorescence intensity of the bleached area was normalized to the fluorescence intensity of the unbleached area, and the fluorescence recovery rate was calculated. For each Hero tobacco analysis, at least four samples from different leaves were examined.

#### Fluorescent imaging

For *Arabidopsis thaliana*, seedlings grown at 22°C for 7–9 days were treated at 42°C for 50 minutes, after which the roots and seedlings were used for particle observation and identification of Hero7-and Hero45-GFP. For tobacco, plants grown at 22°C for 3–4 weeks were treated at 42°C for 50 minutes, and the leaves were used for observation and identification. For *Arabidopsis* protoplasts, Hero7-and Hero45-GFP and DCP1- and PAB8-RFP were co-transformed and incubated in the dark for 16 hours, followed by treatment at 40°C for 50 minutes. GFP was excited at 488 nm and detected in the range of 500–540 nm. RFP was excited at 560 nm and detected in the range of 570–620 nm. To avoid cross-contamination between GFP and RFP signals, the two were imaged sequentially.

### Quantification and statistical analysis

Three biological replicates were performed for phenotypic analysis under normal growth conditions and stress conditions. Relative gene expression values were measured by qRT-PCR and calculated using *Actin7* as a reference. Error bars indicate the standard deviation of the three replicates. Statistical analyses were conducted in accordance with standard protocols established in GraphPad Prism software, employing Student’s t-test for data analysis. The following definition was employed to establish significance levels: The results of the statistical analysis revealed that the values of ns (not significant) were greater than or equal to 0.05. In addition, the values of ∗*p* < 0.05, ∗∗*p* < 0.01, ∗*p* < 0.005, and ∗∗∗∗*p* < 0.001 were observed.
