# Supplementary material for: Heterologous expression of heat-resistant obscure (Hero) proteins enhances thermotolerance in plants
Source: iScience. 2025 Jul 30;28(9):113249. doi: 10.1016/j.isci.2025.113249 (PMC12362396; doi:10.1016/j.isci.2025.113249)
Supplement: Document S1. Figure S1–S7 and Table S1 [file mmc1.pdf]

iScience, Volume *28*

Supplemental information

Heterologous expression of heat-resistant obscure (Hero) proteins enhances thermotolerance in plants

Zhe Kong, Yongping Ke, Heng Zhang, and Daisuke Miki

Supplementary Information

Supplementary Figure Legends

Supplementary Figure S1. Heterologous overexpression of Hero genes in Arabidopsis.

A, Schematic diagram of PcU4-Hero construct.

B, Quantitative mRNA expression analysis. The relative mRNA levels of the indicated genes in six Hero-OE plants were investigated under normal growth condition by using qRT-PCR. The transcript level is calculated relative to *Actin7*; error bars represent the ±SD of triplicate reactions.

Supplementary Figure S2. Growth phenotype of Hero7 and 45 overexpression Arabidopsis.

A, Germination ratio. Data are presented as mean ± SD, n = 3.

B, Bolting timing.

C, Plant height.

D, Flower.

E, Silique size.

F, Silique and fertilization. An analysis was conducted on the plant phenotype of Hero overexpressing plants under normal growth conditions, with the objective of examining the developmental stages. Scale bar = 500 μm.

Supplementary Figure S3. Survival rates of Hero9, 11, 13, and 20 overexpression Arabidopsis.

The survival ratio of the Col-0, Hero9, Hero11, Hero13, and Hero20 seedlings was examined under conditions of heat stress, at a temperature of 44°C for a period of 100 minutes. Data are presented as mean ± SD, n = 3. In each biological replicate, 50 plants per genotype were examined.

Supplementary Figure S4. Mannitol stress tolerance in Hero45 overexpression Arabidopsis.

A, Morphological phenotype of Hero45-OE Arabidopsis seedling. The plants were cultivated in a vertical orientation under standard growth conditions for a period of nine

days, followed by a two-week period in the presence of 300 mM mannitol. Scale bar = 1 cm.

B, Quantitative analysis of the root growth. The root length of Col-0 and Hero45-OE Arabidopsis seedlings (n = 9) was quantified.

C, Quantitative analysis of the chlorophyll content. A Student's *t*-test was employed to determine the standard deviation (n = 3). The results are statistically significant with regard to the control sample. **p < 0.01.

Supplementary Figure S5. Impact of stress-inducible Hero7 on stress tolerance.

A, Schematic representation of the RD29A-Hero7 construct.

B, Phenotype of RD29A-Hero7 plants subjected to heat stress conditions. The phenotype of seven-day-old Arabidopsis seedlings of the Col-0, Hero7-OE, and RD29A-Hero7 plants under normal conditions (upper panel) and upon exposure to a heat stress treatment (lower panel) is presented. The survival rates of the Col-0, Hero7-OE, and RD29A-Hero7 seedlings following the heat treatment were calculated. A Student's *t*-test was employed to determine the standard deviation (n = 3).

C, Morphological phenotype of RD29A-Hero7 plants under mannitol stress conditions. The plants were cultivated in a vertical arrangement under normal conditions (9 days) and mannitol stress conditions (300 mM for 3 weeks). Scale bar = 1 cm. The root length of Col-0, Hero7-OE, and RD29A-Hero7 Arabidopsis seedlings was quantified (upper graph). The chlorophyll content was examined (lower graph). A Student's *t*-test was employed to determine the standard deviation (n = 3). Statistical significance with regard to the control sample is indicated. *p < 0.05.

Supplementary Figure S6. Impact of chloroplast membrane-localized Hero45 on stress tolerance.

A, Schematic representation of the PcU4-Shsp21-Hero construct.

B, Phenotype of chloroplast-membrane-localized Hero45 plants subjected to heat stress conditions. The phenotype of 7-day-old Arabidopsis seedlings of the Col-0 and Shsp21-Hero45 plants under normal conditions (upper panel) and upon exposure to a heat stress treatment (lower panel) is presented. The survival rates of the Col-0 and Shsp21-Hero45 seedlings following the heat treatment were calculated. A Student's *t*-test was used to determine the standard deviation (n = 3).

Supplementary Figure S7. Prediction of disordered status of the Hero7 and Hero45 proteins.

The disorder status of the Hero7 and Hero45 proteins was predicted by IUPred using the default settings. GFP was utilized as a control.

Supplementary Table S1. Sequences of primers used in this study.

Primer name and use	Primer sequence (5' to 3')
**Construction for overexpression**	
Hero7-F	ATGACCCGCGGTAACCAGC
Hero7-R	TTACTTGGGTTCCTCCTTCTTCTCG
Hero9-F	ATGTCGGGCCCCAACGG
Hero9-R	TTAGGGGCTGGCATCACTGG
Hero11-F	ATGGCGCAGGGGCAGC
Hero11-R	TTAGGAAGGTGTCTTGGAGGAGGTG
Hero13-F	ATGGCGAACATCCACCAGGAA
Hero13-R	CGGCATCAGGCAAAACTCGTC
Hero20-F	ATGAGTGCTGCCAGAGAGTCTC
Hero20-R	TTATGAACCACTGGATTTAACAAACATCATTTTATAATTGC
Hero45-F	ATGCCTGGCCATCTCCAAGAA
Hero45-R	TTAGGCCAGAGCTGGAAACGC
GFP-F	ATGGTGAGCAAGGGCGAGGA
GFP-R	TTACTTGTACAGCTCGTCCATGCCG
**qRT-PCR**	
Hero7-QF	atgacccgcggtaaccagc
Hero7-QR	ttacttgggttcctccttcttct
Hero9-QF	atgtcgggccccaacggaga
Hero9-QR	agcatggagttgatggcagcgt
Hero11-QF	gcagcgcaagtttcaggcgca
Hero11-QR	tgggagcgataacacgaccgcc
Hero13-QF	atggcgaacatccaccagga
Hero13-QR	agcgaaagttcggcgctaaac
Hero20-QF	atgagtgctgccagagagtc
Hero20-QR	ctttctttcctgcacccataag
Hero45-QF	atgcctggccatctccaaga
Hero45-QR	gcagctttgagcacttcgaac

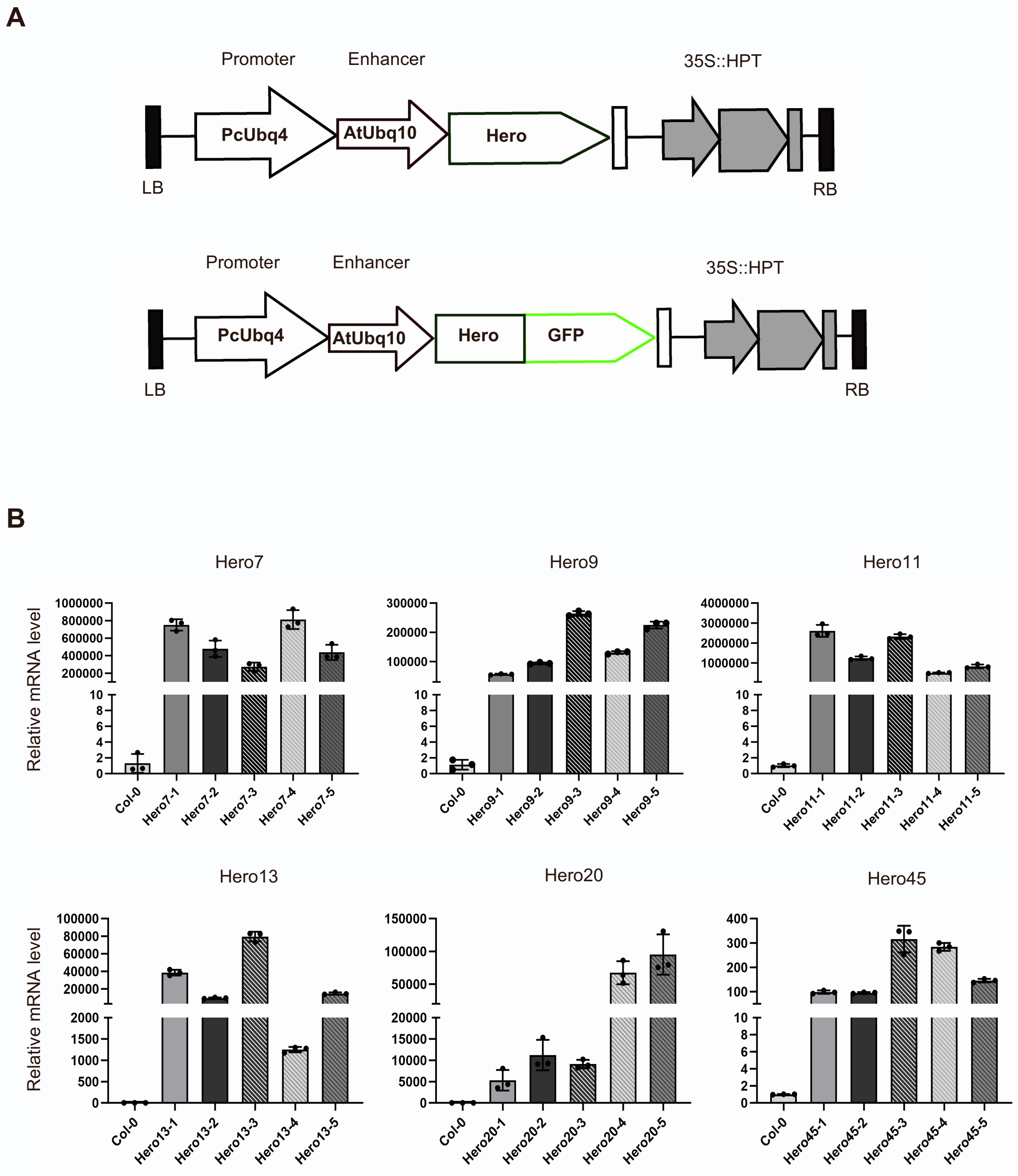

Supplementary Figure S1. Heterologous overexpression of Hero genes in Arabidopsis.

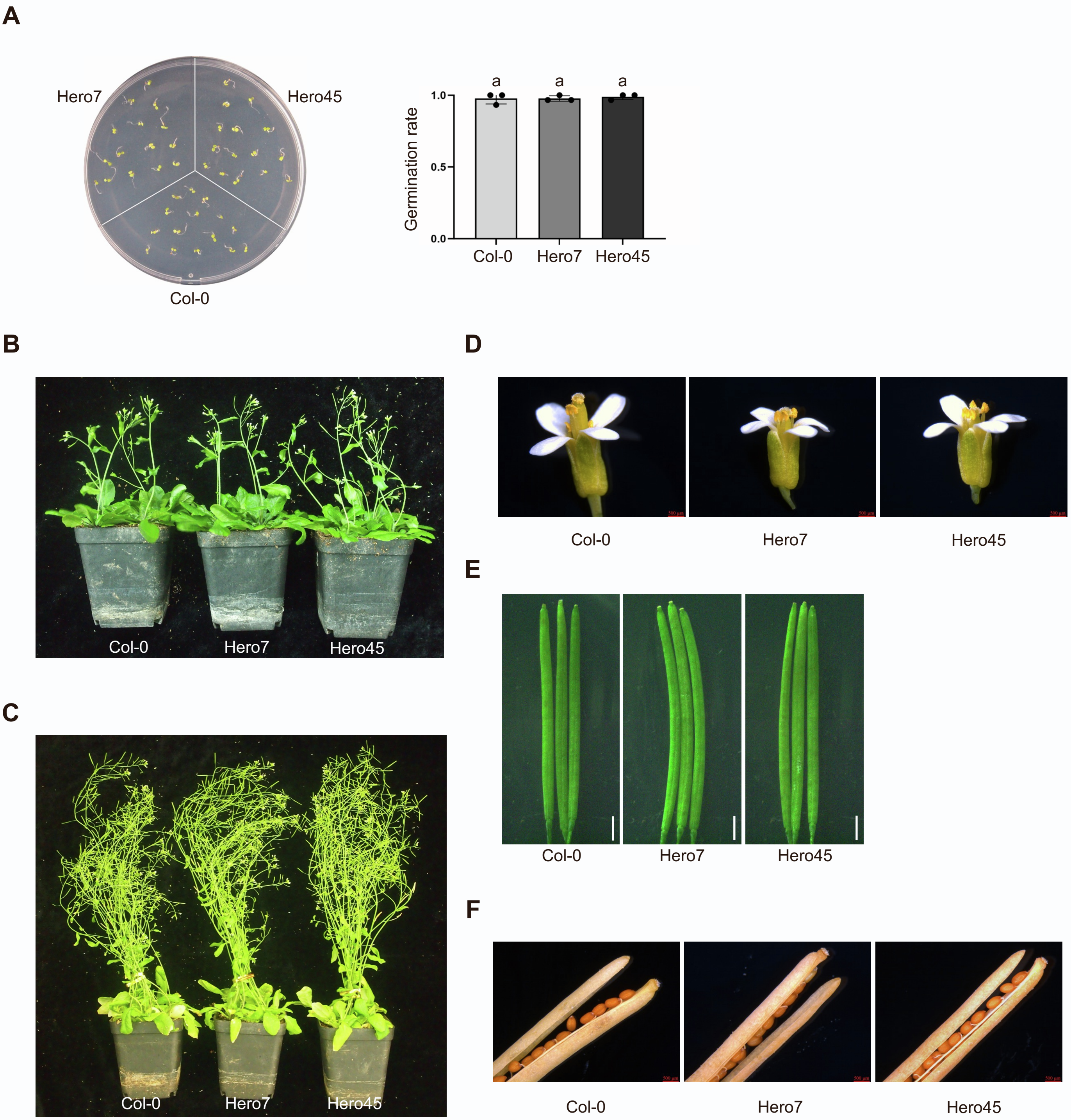

Supplementary Figure S2. Growth phenotype of Hero7 and 45 overexpression Arabidopsis.

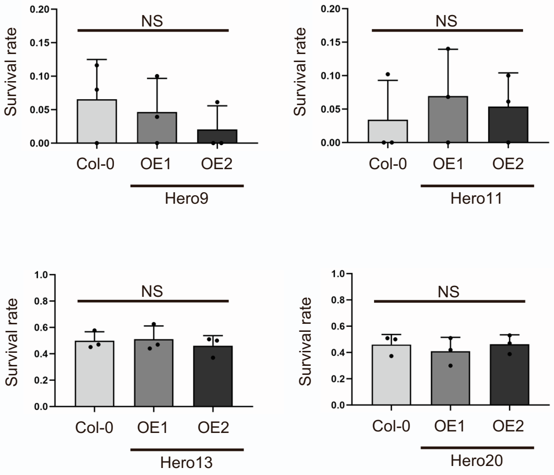

Supplementary Figure S3. Survival rates of Hero9, 11, 13, and 20 overexpression Arabidopsis.

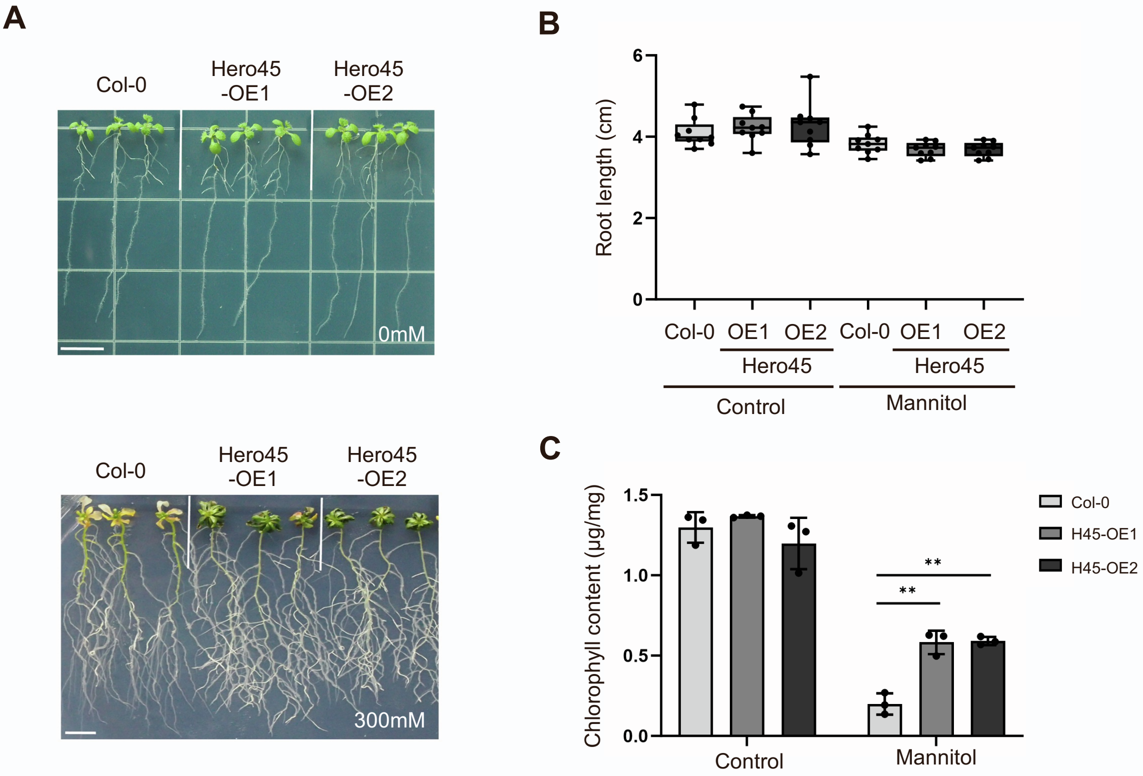

Supplementary Figure S4. Mannitol stress tolerance in Hero45 overexpression Arabidopsis.

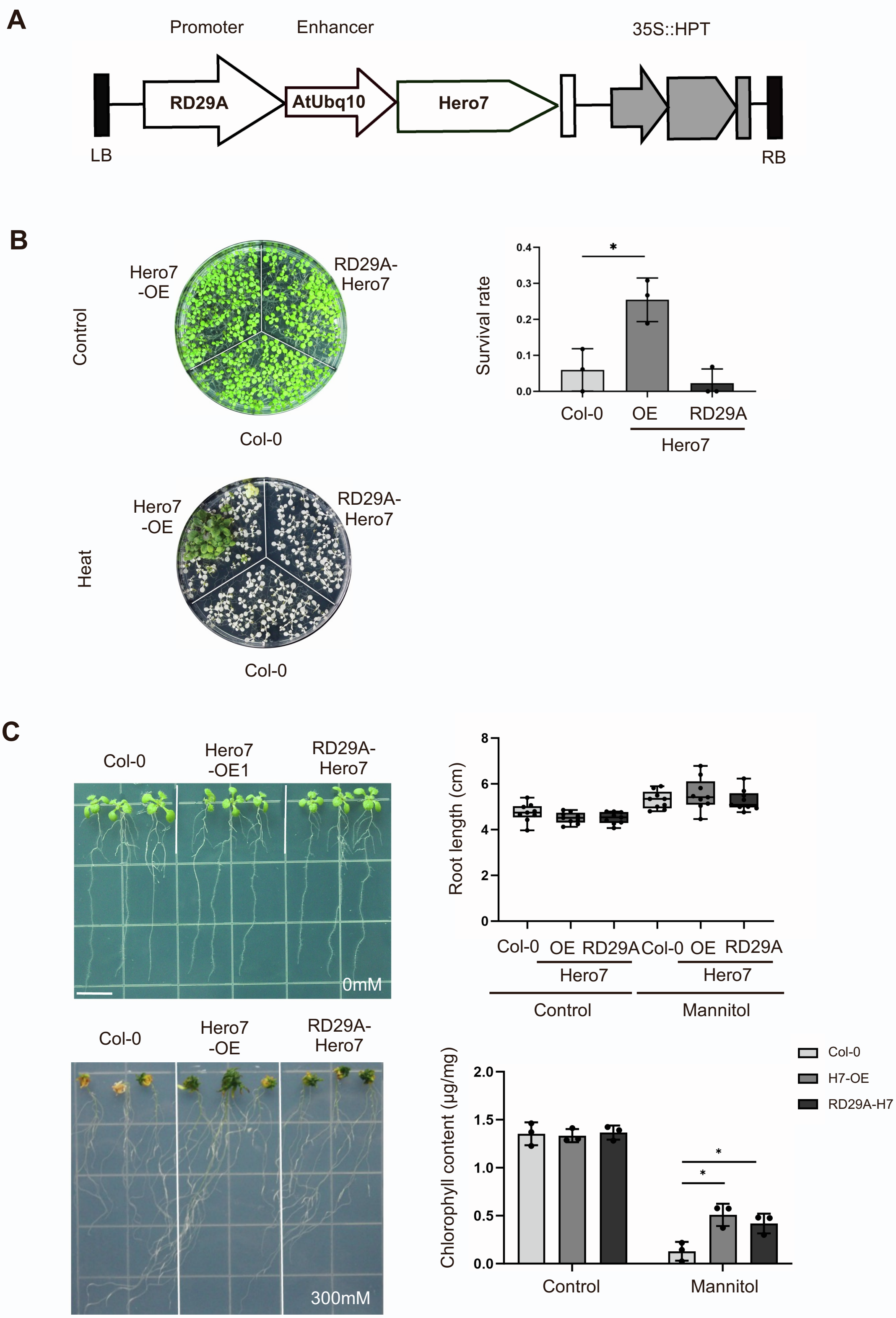

Supplementary Figure S5. Impact of stress-inducible Hero7 on stress tolerance.

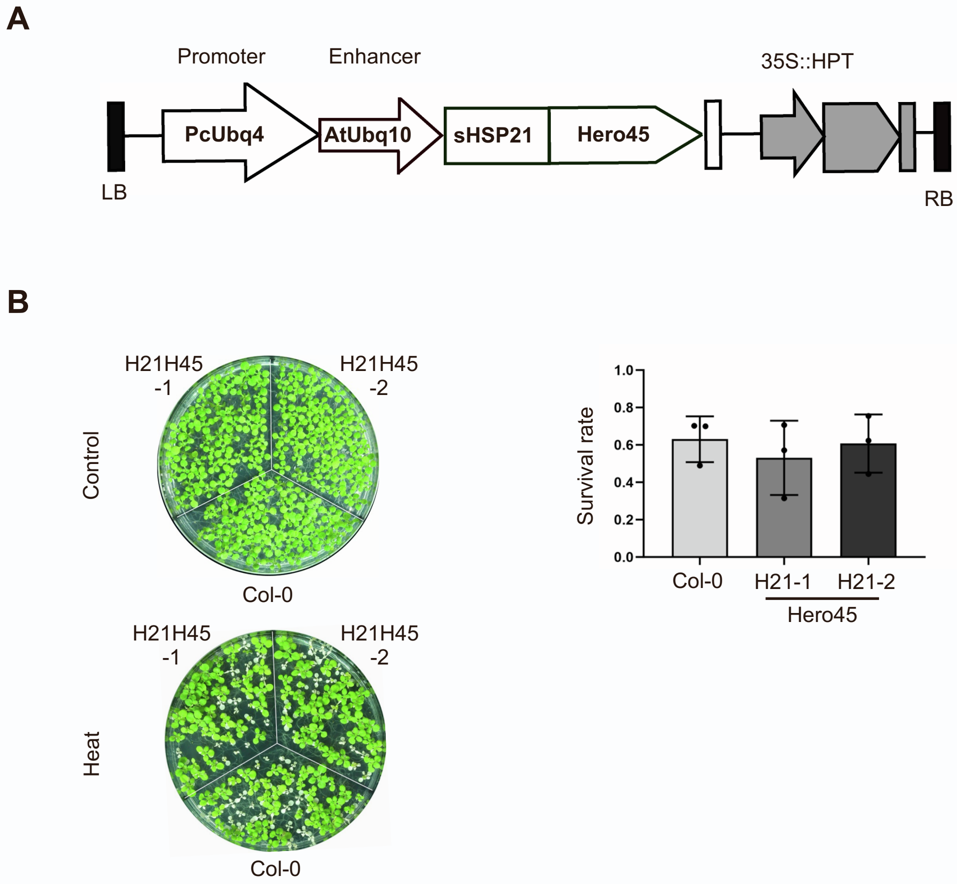

Supplementary Figure S6. Impact of chloroplast membrane-localized Hero45 on stress tolerance.

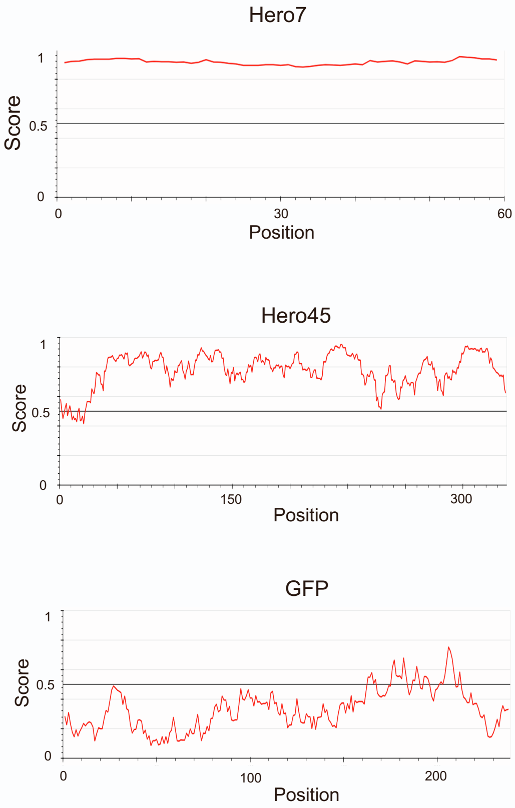

Supplementary Figure S7. Prediction of disordered status in Hero7 and Hero45.
